# mRNA expression pattern of selected candidate genes differs in bovine oviductal epithelial cells in vitro compared with the in vivo state and during cell culture passages

**DOI:** 10.1186/s12958-016-0176-7

**Published:** 2016-08-15

**Authors:** Sadjad Danesh Mesgaran, Jutta Sharbati, Ralf Einspanier, Christoph Gabler

**Affiliations:** Institute of Veterinary Biochemistry, Freie Universität Berlin, Oertzenweg 19b, 14163 Berlin, Germany

**Keywords:** Cell culture passage, Culture medium, Enzymes of cellular metabolism, mRNA expression, Mucin, Oviduct, Prostaglandin synthase

## Abstract

**Background:**

The mammalian oviduct provides the optimal environment for gamete maturation including sperm capacitation, fertilization, and development of the early embryo. Various cell culture models for primary bovine oviductal epithelial cells (BOEC) were established to reveal such physiological events. The aim of this study was to evaluate 17 candidate mRNA expression patterns in oviductal epithelial cells (1) in transition from in vivo cells to in vitro cells; (2) during three consecutive cell culture passages; (3) affected by the impact of LOW or HIGH glucose content media; and (4) influenced by different phases of the estrous cycle in vivo and in vitro. In addition, the release of a metabolite and proteins from BOEC at two distinct cell culture passage numbers was estimated to monitor the functionality.

**Methods:**

BOEC from 8 animals were isolated and cultured for three consecutive passages. Total RNA was extracted from in vivo and in vitro samples and subjected to reverse transcription quantitative polymerase chain reaction to reveal mRNA expression of selected candidate genes. The release of prostaglandin E_2_ (PGE_2_), oviduct-specific glycoprotein 1 (OVGP1) and interleukin 8 (IL8) by BOEC was measured by EIA or ELISA after 24 h.

**Results:**

Almost all candidate genes (prostaglandin synthases, enzymes of cellular metabolism and mucins) mRNA expression pattern differed compared in vivo with in vitro state. In addition, transcription of most candidate genes was influenced by the number of cell culture passages. Different glucose medium content did not affect mRNA expression of most candidate genes. The phase of the estrous cycle altered some candidate mRNA expression in BOEC in vitro at later passages. The release of PGE_2_ and OVGP1 between passages did not differ. However, BOEC in passage 3 released significantly higher amount of IL8 compared with cells in passage 0.

**Conclusion:**

This study supports the hypothesis that candidate mRNA expression in BOEC was influenced by transition from the in vivo situation to the new in vitro environment and during consecutive passages. The consequence of cell culture passaging on BOEC ability to release bioactive compounds should be considered.

## Background

The mammalian oviduct is the site of gamete maturation including sperm capacitation, fertilization, and development of the early embryo [1, 2]. Oviductal epithelial cells play an important role in these reproductive events. In addition, the epithelium layer of the oviduct, which is covered at its apical site with a mucosal surface containing mucins (MUC), provides a physical barrier against invading pathogens [3, 4]. These MUC are grouped as either cell-surface (MUC1, −4, −16) or gel-forming (MUC6) ones [5]. The epithelial cells as the first defense line are also involved in the innate immune response against pathogens. After pathogen recognition through Toll-like receptors [6], epithelial cells release several chemokines, e.g. interleukin 8 (IL8), which are able to attract immune cells to the infected site generating an inflammatory response against microbes [7]. Additionally, oviduct-specific glycoprotein 1 (OVGP1), also named MUC9, is secreted into the lumen of the oviduct and interacts with the gametes assuring the success of fertilization in different species [8].

To fulfill their physiological functions, oviductal epithelial cells can utilize different metabolic pathways as energy source: glycolysis, citric acid cycle, and finally the respiratory chain. Key enzymes of the glycolysis are hexokinase (HK) and glyceraldehyde 3-phosphate dehydrogenase (GAPDH). Especially higher HK activity was observed in the region of the presumptuous fertilization site compared with other regions [9]. An important enzyme as part of the citric acid cycle as well as of the respiratory chain is the succinate dehydrogenase complex, subunit A (SDHA). Ketone bodies are used as a source of energy especially during an energy deficit situation in the cow [10]. Oviductal cells are also supposed to use ketone bodies utilizing specific enzymes as 3-hydroxybutyrate dehydrogenase (BDH2) and 3-oxoacid CoA transferase (OXCT2).

The generated energy is used e.g. for the synthesis of several prostaglandins (PG), which are important for oviduct muscle layers contraction and relaxation supporting the transportation of gametes [11]. Particularly, PGE_2_ release by epithelial cells was stimulated by the presence of sperm and contributes to female’s immunosuppressive effects towards the existing sperm [12]. The PGE_2_ synthesis is conducted starting from the arachidonic acid by the key enzymes PG-endoperoxide synthase 1 and 2 (PTGS1 and −2) to generate PGH_2_, which is converted by specific synthases (PTGES1-3) into PGE_2_ [13].

In order to better understand the abovementioned physiological events, various cell culture models for primary bovine oviductal epithelial cells (BOEC) were established in the last decades [14–16]. Cell monolayer culture models are widely used to elucidate the interactions of the oviduct with spermatozoa [17] or cumulus-oocyte complexes [18] and as feeder cells for embryo development in vitro [19].

Primary cultures resemble cells, which are closer to the tissue origin present in vivo than permanent cell lines. Transcriptome data indicated significant divergence of cell lines from their initial primary cells [20, 21]. In addition, number of passaging strongly effects the mRNA expression of porcine oviductal cells [22] and of human synovial fibroblasts [23]. In this context, cell culture passaging also influences cellular responses by significantly reducing the expression rate of different proteins in synovial and gingival fibroblast cells [24, 25].

Therefore, in vitro conditions should be standardized to achieve the in vivo state of cells as closely as possible. Culture medium plays a crucial part providing proper source of energy and compounds supporting cell growth in vitro. Glucose content should be applied similar to the plasma concentrations of the animal restoring cells in normal condition, because conventional high-glucose medium may be induce undesirable metabolic stress as observed in primary cultured rat myocardial cells [26].

This leads to the important question, how mRNA expression of cultured primary BOEC is perturbed during different numbers of in vitro culturing passages. The groups of investigated candidate genes are (a) mucins and IL8 as parts of the defense system against pathogens, (b) enzymes of cellular metabolism, and (c) key enzymes of the PGE_2_ synthesis. Therefore, the aim of the present study was to evaluate the mRNA expression pattern of these candidate genes in bovine oviductal cells (1) in transition from in vivo state to the in vitro situation in the same animals; (2) monitoring during three consecutive passages; (3) determining the effect of two different glucose concentrations in the cell culture medium; and (4) elucidating the effect of the estrous cycle phase in vivo and in vitro. In addition, the release of a metabolite (PGE_2_) and proteins (OVGP1 and IL8) was determined from BOEC at two distinct cell culture passage numbers. This was primarily done to reflect changes on the mRNA level also on the protein level.

## Methods

### Collection and classification of bovine oviducts

The procedure of oviduct collection and oviductal epithelial cell isolation was performed as previously described [27]. Briefly, both oviducts from non-pregnant Holstein cows (*n* = 8) were collected at the abattoir, transported on ice to the laboratory and processed within 3 h after animal death. Samples from reproductive tracts with signs of inflammation or anatomic defects were not considered. The stage of the estrous cycle was determined based on the assessment of the ovaries (presence of follicle or corpus luteum) along with the evaluation of the uterus and cervix appearance [28, 29]. The oviducts were classified into the following phases of the estrous cycle (two animals per each phase): post-ovulatory (Day 1 to 5), early-to-mid luteal (Day 6 to 12), late luteal (Day 13 to 18), and pre-ovulatory (Day 19 to 21). Oviductal epithelial cells were collected by gently scraping the luminal surface of the opened oviducts with a rubber policeman. Scraped cells from each oviduct were flushed with 1 ml Dulbecco’s phosphate-buffered saline without Ca^2+^/Mg^2+^ (PBS; PAA, Cölbe, Germany) into a 1.5 ml reaction tube, centrifuged at 570 g for 5 min, and the supernatant was removed. Then, cells from both oviducts of each animal were combined by suspending them with 1 ml PBS. A small aliquot of this cell suspension (200 μl) was removed, centrifuged at 570 g for 5 min, and the resulting pellet immediately stored at −80 °C for further analysis representing the in vivo samples. The remaining suspended cells were used for cell culturing.

### Cell culture and passaging

The combined oviductal epithelial cells of one cow were washed twice and suspended with Dulbecco’s Modified Eagle Medium (DMEM) containing 10 % dialyzed fetal calf serum (FCS) (Sigma, Seelze, Germany), 1 g/l (5.5 mM) glucose, 100 U/ml penicillin, 100 μg/ml streptomycin, 50 μg/ml gentamicin and 1 μg/ml amphotericin B (all Biochrom, Berlin, Germany) along with 10 μg/ml reduced glutathione and 10 μg/ml ascorbic acid as antioxidants (both Sigma). Subsequently, 5 ml of the obtained cell suspension (approximately 1–2 × 10^5^ cells/ml) were seeded in six-well plates at 37 °C in a humidified atmosphere of 5 % CO_2_. The abovementioned culturing medium represents the LOW glucose medium. Dialyzed FCS was supplemented to the LOW glucose medium in order to avoid an external additional source of glucose in the medium for cultured cells.

Additionally, HIGH glucose culturing medium was also applied within this study, which contained 4.5 g/l (25 mM) glucose and was supplemented with superior FCS (Biochrom). The other supplements of the medium contents were the same as for the LOW glucose medium. Cultured cells were considered as primary bovine oviductal epithelial cells (BOEC) at passage 0 cultured in LOW or HIGH glucose culture medium, named in the following P0-LOW or P0-HIGH, respectively. Monolayer formation (>80 % confluence) was reached 5 days after cell seeding and cells were detached using accutase (Sigma) until single cells appeared. Detached cells were then subjected to successive subculturing and seeded at a density of 5 × 10^5^ cells/well in 6-well plates in 5 ml of the abovementioned culture medium with LOW or HIGH glucose concentration for the next three passages (P1-LOW or P1-HIGH; P2-LOW or P2-HIGH; P3-LOW or P3-HIGH). Monolayer of >80 % confluence was achieved 72 h after subculturing in the passages 1, 2, and 3.

### Cytokeratin immunofluorescence staining

The purity and homogeneity of each passage were evaluated by staining oviductal cells with a monoclonal mouse anti-human cytokeratin antibody as a marker for epithelial cells (DakoCytomation, Glostrup, Denmark) as previously described [30] with some modifications. Briefly, cells at each passage were cultured with the density of 1.25 × 10^5^ cells/well in multi-well chamber slides (Sarstedt, Nümbrecht, Germany). After reaching confluence, cells were rinsed with PBS (PAA) and fixed using ice cold acetone (Merck, Darmstadt, Germany) for 10 min. After treatment with PBS/1 % bovine serum albumin (Sigma) (blocking medium) for 1 h to block non-specific binding-sites, cells were incubated with the anti-cytokeratin antibody (1.2 μg/ml in blocking medium) for 1 h at room temperature. Cells were washed once for 30 s and two times each for 10 min with PBS. Afterwards, samples were incubated with the goat anti-mouse-IgG secondary antibody conjugated with DyLight 488 (Bio-Rad AbD Serotec, Puchheim, Germany; 1.2 μg/ml in blocking medium) for 1 h at room temperature in the dark. The nuclei were stained with 4′,6-diamidino-2-phenylindole (DAPI; Roche, Mannheim, Germany; 200 ng/ml in PBS) followed by three washing steps with PBS. Finally, slides were mounted with mounting medium (50 % glycerol in PBS) and viewed under a Leica epifluorescence microscope (Leica DMI 6000 B, Wetzlar, Germany). The exposure length and gain remained constant for all samples by using the excitation and emission wavelengths at 494 nm and 519 nm, respectively. Negative controls were performed by substituting the primary antibody with the secondary antibody.

### Wheat germ agglutinin staining

The lectin wheat germ agglutinin (WGA) binds to N-acetyl-D-glucosamine and sialic acid residues [31]. These monosaccharides are part of mucins [32], which makes WGA suitable as an indirect indicator for the presence of MUC on the surface of epithelial cells [33].

The procedure of WGA staining for paraffin embedded oviduct sections and cultured BOEC was carried out as described by manufacturer’s instructions (Invitrogen, Darmstadt, Germany).

In brief, oviductal cross-sections of 3–5 μm from each estrous cycle stage were adhered to SuperFrost Plus microscope slides, fixed overnight at 56 °C, and stored until use at room temperature [27]. In order to liquefy the paraffin, slides were heated for 60 min at 60 °C. Afterwards, sections were immediately deparaffinized in xylene, rehydrated through graded ethanol, rinsed with deionized water, and washed with PBS for 10 min. Subsequently, slides were incubated with WGA Alexa Fluor 594 conjugate [Invitrogen, 5 μg/ml in Hank’s balanced salt solution (HBSS; Biochrom)] for 15 min at room temperature in the dark. Slides were washed twice with HBSS and nuclei were stained with DAPI (200 ng/ml in PBS) for 10 min. Finally, tissues were rinsed, washed once for 10 min with PBS, and mounted with mounting medium (50 % glycerol in PBS). Pictures were taken with a Leica epifluorescence microscope (Leica DMI 6000 B). The exposure length and gain remained constant for all samples by using the excitation and emission wavelengths at 590 nm and 617 nm, respectively.

Oviductal cells of each passage with the density of 1.25 × 10^5^ cells/well were cultured in multi-well chamber slides (Sarstedt) until confluence. Afterwards, cells were fixed with 4 % formaldehyde for 15 min. Following three times washing with HBSS, cells were incubated with WGA Alexa Fluor 594 conjugate (5 μg/ml in HBSS) for 10 min at room temperature in the dark. Cells were washed once for 30 s and two times each for 10 min with PBS and nuclei were stained with DAPI (200 ng/ml in PBS) for 10 min. Finally, slides were rinsed, washed twice with PBS, and mounted with mounting medium (50 % glycerol in PBS) to view under a Leica epifluorescence microscope with the same conditions as described above.

### Total RNA extraction and cDNA synthesis

Total RNA from oviductal cell pellets representing the in vivo samples was extracted using the NucleoSpin RNA kit (Macherey-Nagel, Düren, Germany) following the manufacturer’s instructions. Cultured oviductal cells after reaching >80 % confluence at each passage were subjected to total RNA isolation performed with the InviMag Universal RNA Mini Kit (Stratec Molecular, Berlin, Germany) using the KingFisherFlex (Thermo Scientific, Langenselbold, Germany) according to the manufacturer’s instructions with a slight modification. Cells were lysed with buffer RLT (Qiagen, Hilden Germany) supplemented with 1 % (*v/v*) 2-mercaptoethanol (Sigma) immediately after medium removal within the culture plates. Quantification of the extracted total RNA was done photometrically at 260 nm using NanoDrop ND-1000 UV–VIS spectrophotometer (Peqlab Biotechnologie, Erlangen, Germany). The integrity of total RNA was determined by using RNA 6000 Nano Chip on an Agilent 2100 Bioanalyzer (Agilent Technologies, Waldbronn, Germany).

Reverse transcription (RT) of 0.25 μg total RNA was carried out in a total volume of 60 μl as previously described [27]. Complementary DNA was synthesized using 200 U RevertAid Moloney-Murine Leukaemia Virus Reverse Transcriptase, 2.5 μM random hexamer primers, 0.66 mM dNTPs, and 1x of the supplied RT buffer (all Fermentas, St. Leon-Rot, Germany). DNA digestion was performed before RT in order to remove genomic DNA contaminations [34]. Samples without the reverse transcriptase were included to confirm the absence of any genomic DNA or other contaminations. The synthesized cDNA was stored in aliquots at −20 °C until use.

### Quantitative PCR

Quantification of mRNA expression was performed as previously described [27] according to minimum information for publication of quantitative real-time PCR experiments (MIQE) guidelines [35]. Each reaction with a total volume of 10 μl contained 1 μl cDNA, 0.4 μM of forward and reverse primer each (details are given in Tables 1 and 2; synthesized by Eurofins MWG, Ebersberg, Germany), and 1x SensiMix SYBR HI-ROX (Bioline, Luckenwalde, Germany). The amplification was done by a StepOne Plus device (Applied Biosystems, Darmstadt, Germany) and performed with an initial denaturation at 95 °C for 10 min, followed by 45 cycles of denaturation at 95 °C for 15 s, annealing at temperature indicated in Tables 1 and 2 for 20 s, and extension at 72 °C for 30 s. After amplification, samples were heated at the rate of 0.5 °C/15 s from 50 to 99 °C with continuous reading of fluorescence to obtain a melting curve followed by a final step of cooling to 40 °C. Negative controls by substituting template DNA with water and samples without RT enzyme were also included in each run.

**Table 1:** Primer pairs for amplification of genes of interest

Gene	Sequence of nucleotide	Accession no. /Reference	Product size (bp)	Tm (°C)
PTGS1	for 5′ -CAG ATG CGG AGT TTC TGA GTC G- 3′	[27]	313	60
	rev 5′ -GGG TAG TGC ATC AGC ACG G- 3′			
PTGS2	for 5′ -CTC TTC CTC CTG TGC CTG AT- 3′	[27]	359	60
	rev 5′ -CTG AGT ATC TTT GAC TGT GGG AG- 3′			
PTGES2	for 5′ -CCT CCT ACA GAA AGG TGC C- 3′	[56]	133	56
	rev 5′ -GTG ATG ATG TCT GCC AGG G- 3′			
PTGES3	for 5′ -TGC AAA GTG GTA CGA TCG G- 3′	[56]	253	61
	rev 5′ -TAA CCT TGG CCA TGA CTG G- 3′			
MUC1	for 5′ -ATG ACC ACC CGC TCT ATG TC- 3′	AJ400824	189	60
	rev 5′ -GGA GGT GGA AAG TGC TAT GC- 3′			
MUC4	for 5′ -ACG TCA CTG TGC ATC TTT GG- 3′	XM_002684819.3	199	60
	rev 5′ -AAG CTC TTG ATG GAC GGT TG- 3′			
MUC6	for 5′ -CAG CAG TCC CAC TTC CTC TG- 3′	XM_005197398.3	206	65
	rev 5′ -CAG TGA TGG AGC TGG CTA GG- 3′			
MUC16	for 5′ -CAG GTC TCA AAA TCC CAT CC- 3′	XM_002688785.3	256	62
	rev 5′ -TGC TGG AGG TGT TGA TAT GG- 3′			
OVGP1	for 5′ -GGG AAA GGT TCG TCA GTT CA- 3′	NM_001080216.1	240	60
	rev 5′ -CAT ACG CTT TCT GGA CGA CA- 3′			
BDH2	for 5′ -ATG TCC TCT GTG GCT TCC AG- 3′	NM_001034488.2	347	59
	rev 5′ -CAC AAA CTC CAG CCT CCA TC- 3′			
OXCT2	for 5′ -CAC AGT GAG AAC GGG ATC TTG- 3′	XM_002704022.2	347	55
	rev 5′ -GTG CAC TTC TCC ACG ATC TTG- 3′			
GAPDH	for 5′ -CCC AGA AGA CTG TGG ATG G- 3′	[57]	306	62
	rev 5′ -AGT CGC AGG AGA CAA CCT G- 3′			
SDHA	for 5′ -GGG AGG ACT TCA AGG AGA GG- 3′	[57]	219	60
	rev 5′ -CTC CTC AGT AGG AGC GGA TG- 3′			
HK1	for 5′ -GCG TTT CCA CAA GAC TCT GC- 3′	XM_010820525	324	61
	rev 5′ -AGA TCC AGG GCC AAG AAG TC- 3′			
IL8	for 5′ -CGA TGC CAA TGC ATA AAA AC- 3′	[58]	153	56
	rev 5′ -CTT TTC CTT GGG GTT TAG GC- 3′			

Selected gene transcripts, primer sequences and annealing temperatures (Tm) used for quantitative PCR with resulting amplicon length

**Table 2:** Primer pairs for amplification of reference genes

Gene	Sequence of nucleotide	Accession no. /Reference	Product size (bp)	Tm (°C)
HDAC1	for 5′ –CCA GTG CAG TTG TCT TGC AG- 3′	NM_001037444.2	217	60
	rev 5′ –TTA GGG ATC TCC GTG TCC AG- 3′			
UXT	for 5′ –CGC TAC GAG GCT TTC ATC TC- 3′	NM_001037471.2	207	61
	rev 5′ –TGA AGT GTC TGG GAC CAC TG- 3′			
PPIA	for 5′ – CTG AGC ACT GGA GAG AAA GG- 3′	NM_178320.2	259	60
	rev 5′ – TGC CAT CCA ACC ACT CAG TC- 3′			
RPL19	for 5′ -GGC AGG CAT ATG GGT ATA GG- 3′	NM_001040516.1	232	60
	rev 5′ -CCT TGT CTG CCT TCA GCT TG- 3′			
SUZ12	for 5′ -TTC GTT GGA CAG GAG AGA CC- 3′	[59]	286	60
	rev 5′ -GTG CAC CAA GGG CAA TGT AG- 3′			

Selected gene transcripts, primer sequences and annealing temperatures (Tm) used for normalization of quantitative PCR with resulting amplicon length

Serial dilutions of the appropriate purified amplicons were used as a standard curve for gene quantification. Standards were generated as previously described [27]. Besides the melting curve, the obtained amplicons were checked for specificity by sequencing (GATC Biotech, Konstanz, Germany) and showed a 100 % homology to the published bovine sequences.

### ELISA and EIA

Medium from oviductal cells of P0-HIGH and P3-HIGH cultured in 24-well plates was removed after reaching confluence. Cells were incubated with 0.5 ml DMEM HIGH glucose culture media at 37 °C in a humidified atmosphere of 5 % CO_2_. After 24 h, the supernatant was collected and stored at −80 °C for further analysis. Prior to each assay, samples were centrifuged at 13,000 g for 5 min to pellet any dead/floating cells remaining in the supernatants. Duplicates of each sample (100 μl) were used to estimate in a 96 well microtiter plate the release of PGE_2_ (Parameters kit; R&D Systems, Wiesbaden, Germany), OVGP1 (MyBioSource, San Diego, USA) and IL8 (Human CXCL8/IL8 antibody DuoSet; R&D Systems) from BOEC using commercially available kits. Every assay was conducted based on the manufacturer’s instructions. Samples were three-fold diluted for the PGE_2_ assay with the same BOEC culture medium. Previous work confirmed the cross-reactivity of human CXCL8/IL8 antibodies with bovine IL8 [36]. All assays were carried out using BOEC culture supernatants from different cows (IL8: *n* = 4; PGE_2_: *n* = 3; OVGP1: *n* = 3). Amounts of the mentioned factors in supernatants were evaluated with a microtiter reader (iMark Bio Rad, Bio-Rad Laboratories, Munich, Germany) after optical density measurements at 450 nm based on the generated standard curve. The range of standard curve for PGE_2_, IL8 and OVGP1 were 15.6–2000 pg/ml, 39–2500 pg/ml and 0–1000 ng/ml, respectively.

### Statistical analysis

GeNorm tool [37], which calculates a normalization factor based on the geometric mean of expression levels of reference genes, was assessed to normalize the sample to sample variation of the mRNA expression data. Initially, real-time PCR data of histone deacetylase 1 (HDAC1), ubiquitously-expressed transcript (UXT), peptidylprolyl isomerase A (cyclophilin A) [PPIA], suppressor of zeste 12 homolog (SUZ12) and 60S ribosomal protein L19 (RPL19) were tested with geNorm. Due to their higher expression stability among samples, HDAC1 and UXT were chosen for normalizing mRNA expression of the genes of interest in this study. Normalized data were used for generation of box and whiskers plots with GraphPad Prism 6 (GraphPad Software, La Jolla, USA) displaying median values with 50 % of data within the box. Outliers are shown as circles.

Shapiro-Wilk test was performed to determine the normality of data distribution. Based on the normality test output, either one-factor analysis of variance (ANOVA) or Kruskal-Wallis-Test was performed followed by *post hoc* Tukey or Mann–Whitney *U* test to analyze the effect of passaging. Either *t*-test or Mann–Whitney *U* test was conducted to test the effect of glucose medium content at each cell culture passage. Mann–Whitney *U* test was used to test the effect of estrous cycle phases (luteal phase versus non-luteal phase; *n* = 4 each) on in vivo cells or on cultured BOEC at each number of cell culture passage. Fold changes were calculated as the approximate ratio of the mean of normalized mRNA expression to compare different groups.

Bar charts generated from ELISA data are presented as the mean ± SEM (*n* = 3 cows for OVGP1; *n* = 4 for IL8; *n* = 3 for PGE_2_). Mann–Whitney *U* test was conducted to compare rate of abovementioned contents between P0-HIGH and P3-HIGH cell culture supernatants.

All statistical analyses were performed with SPSS Statistics for Windows Version 20 (SPSS, Chicago, USA) and the level of significance was set at P ≤ 0.05.

## Results

### Cytokeratin staining and morphology

**Fig. 1:**
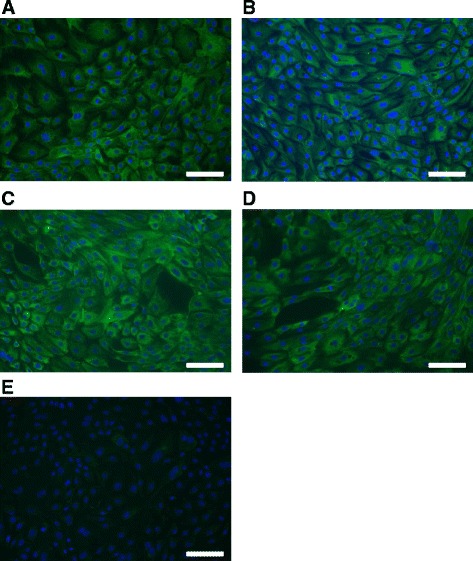
Cytokeratin immunostaining of cultured BOEC in different number of cell culture passages. Immunostaining with anti-cytokeratin antibody of cultured BOEC in: **a** passage 0; **b** passage 1; **c** passage 2; **d** passage 3; and **e** negative control. Goat anti-mouse-IgG DyLight 488 conjugate (green) was used for staining cytokeratin as a secondary antibody. DAPI (blue) was used to visualize nuclei. Magnification was set at 200 X. Bar in each figure represents 100 μm. Representative pictures of BOEC cultured in LOW glucose medium are shown

A purity >99 % of epithelial cells at each passage was determined with cytokeratin immunofluorescence staining as a specific marker for epithelial cells (Fig. 1a-d). Oviductal stromal cell contamination in all in vitro cell culture passages was below 1 %. Furthermore, there was no difference in the purity cultured with either LOW or HIGH glucose medium content at each passage. In the negative control no specific staining for cytokeratin was observed (Fig. 1e).

Cells through all passages within this study retained their epithelial cell heterogeneity. Oviductal cells in P0, P1 and P2 (Fig. 1a, b and c, respectively) had a polygonal structure. However, it was observed that cultured BOEC in P3 showed a tendency towards morphology change to lose this type of structure to appear more elongated (Fig. 1d). There was no evident difference of BOEC morphology with either LOW or HIGH glucose medium at each passage.

### Wheat germ agglutinin staining

**Fig. 2:**
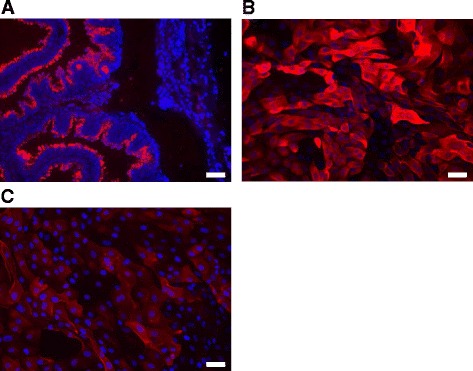
WGA staining in sections of oviduct tissue and cultured BOEC. WGA-Alexa Fluor 594 (red) staining of: **a** oviductal cross-section of the ampulla collected during the luteal phase represents in vivo sample; **b** primary cultured BOEC in passage 0; **c** primary cultured BOEC in passage 3. DAPI (blue) was used to visualize nuclei. Magnification was set at 200 X. Bar in each figure represents 50 μm. Representative pictures of BOEC cultured in HIGH glucose medium are shown

The presence of N-acetyl-D-glucosamine and sialic acid residues as parts of mucins was detected on oviductal cells both in vivo and in vitro by WGA staining (Fig. 2).

In detail, oviductal cells in vivo were positively stained with WGA in samples obtained from the luteal as well as from the non-luteal phase without any obvious differences. A representative picture from the stained section obtained during the luteal phase is presented (Fig. 2a). The WGA staining was observed only at the apical surface of the epithelium lining. The basal surface of the epithelial cells was not stained. In addition, staining could neither be detected on stromal nor on endothelial cells.

Concerning oviductal cells in vitro, WGA staining was observed on the entire surface of cultured cells in all passages. However, the WGA staining of the minimum and maximum number of cell passages (P0 and P3, respectively) showed differences. The staining appeared on more cells in P0 compared with P3 (Fig. 2b and c). In addition, the staining was noted to be stronger on more positive stained cells in P0 than in P3.

### mRNA expression of selected PG synthesis enzymes

**Fig. 3:**
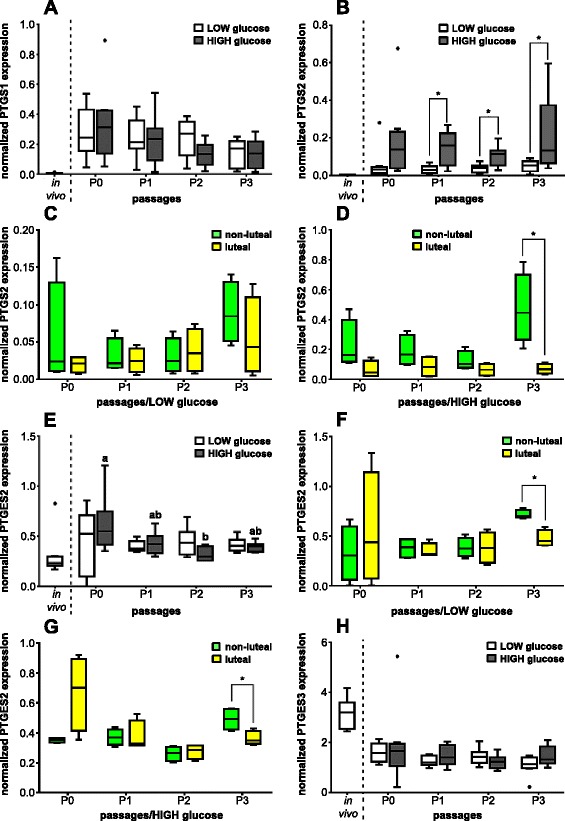
Effect of passage number or estrous cycle stage on PG synthesis enzyme mRNA expression pattern. Normalized mRNA expression of **a** PTGS1; **b** PTGS2; **e** PTGES2; and **h** PTGES3 in BOEC of in vivo samples and of cell culture passages P0, P1, P2 and P3 with LOW and HIGH glucose medium content (*n* = 8) as well as normalized mRNA expression depending of the phase of the estrous cycle on day of collecting cells of **c** PTGS2 in LOW glucose medium; **d** PTGS2 in HIGH glucose medium; **f** PTGES2 in LOW glucose medium; and **g** PTGES2 in HIGH glucose medium (*n* = 4). Different letters indicate significant difference (*P* < 0.05) between cell culture passages of the same glucose medium content. Asterisk on top of the line represents significant difference between different glucose content of medium at each cell culture passage or between the different estrous cycle phases on day of collection (*P* < 0.05)

The mRNA for PTGS1 was about 20-fold more highly expressed in cultured oviductal cells compared with cells obtained from the in vivo state (Fig. 3a). However, PTGS1 mRNA expression was neither affected by the number of cell culture passages nor by the different medium glucose content. In addition, there was no difference of PTGS1 mRNA expression in BOEC obtained at different phases of the estrous cycle cultured with either LOW or HIGH glucose content medium at all passages (data not shown).

Similar as observed for PTGS1, PTGS2 mRNA expression in oviductal cells differed tremendously in the in vitro state with at least tenfold higher contents compared with the in vivo state (Fig. 3b). The glucose content of the culturing medium impacted the mRNA expression pattern of PTGS2. Throughout the passages P1, P2 and P3, BOEC cultured in HIGH glucose medium showed more than threefold higher PTGS2 mRNA expression than their cultured counterparts with LOW glucose medium in the same cell culture passage (*P* < 0.05). Additionally, oviductal cells in P0-HIGH tended to show higher transcript amount for PTGS2 than in P0-LOW cells (*P* = 0.07). In contrast, mRNA expression of PTGS2 was not affected by the number of cell culture passages. The stage of estrous cycle did not affect PTGS2 transcription in BOEC cultured with LOW glucose medium (Fig. 3c), whereas oviductal cells collected around ovulation cultured with HIGH glucose medium showed in P3 a significant sevenfold higher PTGS2 mRNA expression compared with cells harvested during the luteal phase (Fig. 3d).

There was an alteration of PTGES2 transcription in BOEC from the in vivo state to in vitro cells with a higher PTGES2 mRNA content at least after the first passage (Fig. 3e). The expression of PTGES2 mRNA was also influenced by the cell culture passage in the presence of HIGH glucose medium but not with LOW glucose containing medium. The mRNA amount of PTGES2 in P2-HIGH BOEC was significantly lower (*P* < 0.05) compared with cells in P0-HIGH. In contrast, the different content of glucose in the medium did not influence significantly the mRNA expression pattern of PTGES2 (*P* > 0.05). Regardless to the glucose content in media, BOEC obtained around ovulation had significant higher (*P* < 0.05) PTGES2 mRNA abundance in P3 than cells harvested during the luteal phase (Fig. 3f and g).

In opposite to the other PG synthases, oviductal cells in vitro showed approximately twofold lower PTGES3 mRNA expression than in the in vivo state (Fig. 3h). On the other hand, mRNA expression pattern of PTGES3 was neither influenced by the number of cell culture passages nor by the different glucose content of the medium. Furthermore, the phase of estrous cycle did not show any significant effect on the transcription rate of PTGES3 in cultured BOEC with either LOW or HIGH glucose medium (data not shown).

The mRNA expression of all investigated PG synthases in oviductal cells in vivo was not influenced by the phase of estrous cycle grouping them into the non-luteal (around ovulation; *n* = 4) or luteal (*n* = 4) phase (data not shown).

### mRNA expression of selected mucins and IL8

**Fig. 4:**
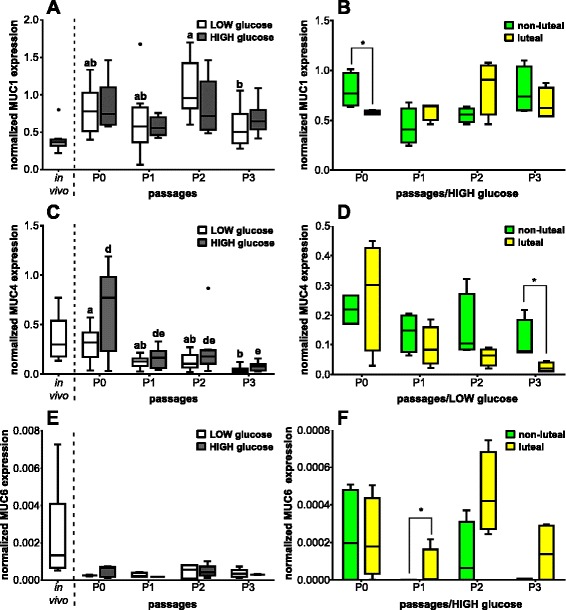
Effect of passage number or estrous cycle stage on selected mucin mRNA expression pattern. Normalized mRNA expression of **a** MUC1; **c** MUC4; and **e** MUC6 in BOEC of in vivo samples and of cell culture passages P0, P1, P2 and P3 with LOW and HIGH glucose medium content (*n* = 8) as well as normalized mRNA expression depending of the phase of the estrous cycle on day of collecting cells of **b** MUC1 in HIGH glucose medium; **d** MUC4 in LOW glucose medium; and **f** MUC6 in HIGH glucose medium (*n* = 4). Different letters indicate significant difference (*P* < 0.05) between cell culture passages of the same glucose medium content (ab and de for LOW and HIGH glucose medium content, respectively). Asterisk on top of the line represents significant difference between the different estrous cycle phases on day of collection (*P* < 0.05)

The mRNA expression of MUC1 was about twofold higher in oviductal cells in vitro compared with cells from the in vivo state (Fig. 4a). There was no significant effect of different glucose concentration in the culturing medium on the MUC1 mRNA expression. However, cell culture passage influenced the mRNA expression pattern of MUC1 (*P* < 0.05) in cells cultured with LOW glucose medium but not with a HIGH glucose medium. Oviductal cells in P2-LOW showed a twofold higher MUC1 mRNA expression than in P3-LOW (*P* < 0.05). The phase of estrous cycle did not impact MUC1 mRNA expression in BOEC cultured with LOW glucose medium (data not shown). However, there was a significant higher (*P* < 0.05) MUC1 mRNA expression in P0-HIGH in oviductal cells harvested during the non-luteal phase compared with oviductal cells collected during the luteal phase (Fig. 4b).

Although oviductal cells in P0 showed similar MUC4 mRNA expression pattern to cells in the in vivo state, an apparent decrease was detected in BOEC at the other passages compared with in vivo cells (Fig. 4c). The transcription rate of MUC4 was influenced by the number of cell culture passages. Cultured cells in P0-LOW and P0-HIGH displayed an approximately tenfold higher MUC4 mRNA expression compared with cultured BOEC in P3-LOW and P3-HIGH, respectively (*P* < 0.05). Different glucose concentration in the medium did not influence MUC4 mRNA expression (*P* > 0.05). The MUC4 transcript amount in BOEC collected around ovulation was significantly fivefold higher (*P* < 0.05) in LOW glucose medium in P3 compared with oviductal cells harvested during the luteal phase (Fig. 4d). In contrast, cells cultured with HIGH glucose medium were not affected by the stage of the estrous cycle in their MUC4 mRNA expression (data not shown).

In general, mRNA expression of MUC6 was the lowest one of all investigated mucins. Bovine oviductal cells showed an approximately tenfold decrease regarding the MUC6 mRNA expression from the in vivo situation to the in vitro state (Fig. 4e). Number of cell culture passages as well as different glucose contents of the medium did not affect MUC6 mRNA expression (*P* > 0.05). The stage of estrous cycle did not impact the LOW glucose cultured oviductal cells (data not shown). However, in oviductal cells collected during the luteal phase cultured with HIGH glucose medium MUC6 mRNA was 40-fold more highly expressed in P1 (*P* < 0.05) than in BOEC harvested during the non-luteal phase (Fig. 4f).

**Fig. 5:**
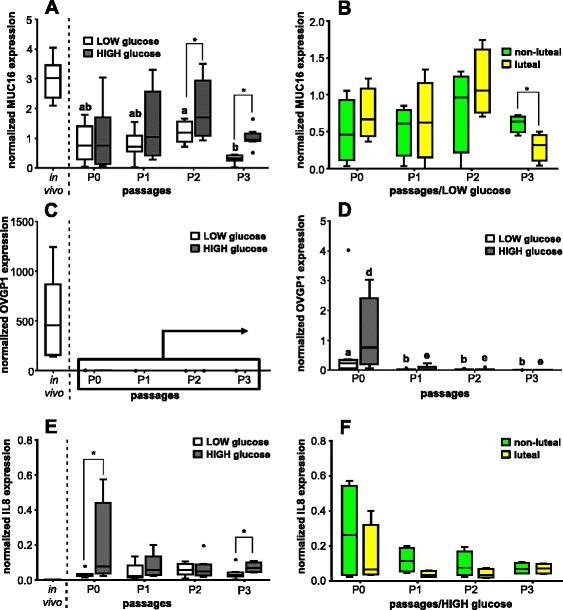
Effect of passage number or estrous cycle stage on MUC16, OVGP1 and IL8 mRNA expression. Normalized mRNA expression of **a** MUC16; **c** OVGP1; and **e** IL8 in BOEC of in vivo samples and of cell culture passages P0, P1, P2 and P3 with LOW and HIGH glucose medium content (*n* = 8); and **d** magnified inset of OVGP1 in BOEC in cell culture passages P0, P1, P2 and P3 with LOW and HIGH glucose medium content as well as normalized mRNA expression depending of the phase of the estrous cycle on day of collecting cells of **b** MUC16 in LOW glucose medium; and **f** IL8 in HIGH glucose medium (*n* = 4). Different letters indicate significant difference (*P* < 0.05) between cell culture passages of the same glucose medium content (ab and de for LOW and HIGH glucose medium content, respectively). Asterisk on top of the line represents significant difference between different glucose content of medium at each cell culture passage or between the different estrous cycle phases on day of collection (*P* < 0.05)

Cultured BOEC displayed a twofold decrease of MUC16 mRNA expression, especially in cells cultured with LOW glucose medium, compared with oviductal cells in vivo (Fig. 5a). The number of cell culture passages concerning LOW glucose containing medium had a significant effect (*P* < 0.05) on the MUC16 mRNA expression with significant fourfold higher contents in cells in P2-LOW than in P3-LOW. In contrast, HIGH glucose medium did not affect the MUC16 mRNA expression. Moreover, different glucose content in the medium influenced the MUC16 mRNA expression revealing in P2-HIGH and P3-HIGH about threefold higher transcription rate of MUC16 than in P2-LOW and P3-LOW cells, respectively (*P* < 0.05). The phase of estrous cycle had a significant impact in P3 on MUC16 mRNA expression in BOEC cultured with a LOW glucose medium (Fig. 5b) but not with a HIGH glucose medium (data not shown). Cultured BOEC obtained during the non-luteal phase had a significant twofold higher (*P* < 0.05) MUC16 mRNA amount in comparison with cells harvested during the luteal phase.

When BOEC were cultured, the OVGP1 mRNA expression decreased tremendously to more than 400-fold lower levels compared with the in vivo state (Fig. 5c). The cell culture passage had also a significant effect (*P* < 0.05) on the OVGP1 mRNA expression pattern in oviductal cells, which in both P0-LOW and P0-HIGH was more than 20-fold higher than in the other passages (Fig. 5d). The different glucose concentration of the medium did not influence significantly OVGP1 mRNA expression (*P* > 0.05). The stage of estrous cycle did not affect OVGP1 transcription in oviductal cells in vitro regardless to the glucose content of media (data not shown).

In addition to the presented candidate mucins, mRNA expression of MUC5AC, −12, and -15 was investigated. Oviductal cells in vivo displayed mRNA expression of these mentioned mucins. However, this was in most samples in vitro near or below the detection limits (data not shown).

Transcript amount of each investigated mucin in oviductal cells in vivo showed no influence by the phase of estrous cycle grouping them into the non-luteal (around ovulation; *n* = 4) or luteal (*n* = 4) phase (data not shown).

Cultured oviductal cells displayed a minimum 40-fold higher expression of IL8 mRNA than in vivo (Fig. 5e). Number of cell culture passages did not affect IL8 mRNA expression pattern. The effect of glucose concentration in the culture medium was apparent in P0 and P3 where cells cultured with HIGH glucose medium in both passages showed higher IL8 mRNA expression (*P* < 0.05) compared with LOW glucose medium cultured cells. The stage of the estrous cycle did not influence the IL8 transcription level of in vivo collected oviductal cells (data not shown). BOEC cultured in P1 with HIGH glucose medium obtained in the non-luteal phase tended (*P* = 0.08) to show higher IL8 mRNA expression than cells collected in the luteal phase (Fig. 5f). The estrous cycle stage did not show any influence on IL8 mRNA abundance in cultured BOEC with LOW glucose content medium (data not shown).

### mRNA expression of selected enzymes of cellular metabolism

**Fig. 6:**
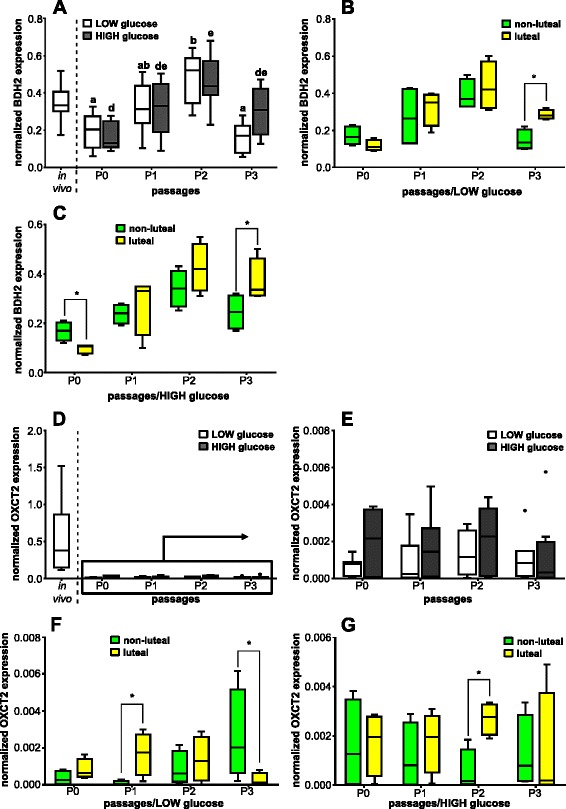
Effect of passage number or estrous cycle stage on enzymes of cellular metabolism mRNA expression. Normalized mRNA expression of **a** BDH2; and **d** OXCT2 in BOEC of in vivo samples and of cell culture passages P0, P1, P2 and P3 with LOW and HIGH glucose medium content (*n* = 8); and **e** magnified inset of OXCT2 in BOEC in cell culture passages P0, P1, P2 and P3 with LOW and HIGH glucose medium content as well as normalized mRNA expression depending of the phase of the estrous cycle on day of collecting cells of **b** BDH2 in LOW glucose medium; **c** BDH2 in HIGH glucose medium; **f** OXCT2 in LOW glucose medium; and **g** OXCT2 in HIGH glucose medium (*n* = 4). Different letters indicate significant difference (*P* < 0.05) between cell culture passages of the same glucose medium content (ab and de for LOW and HIGH glucose medium content, respectively). Asterisk on top of the line represents significant difference between the different estrous cycle phases on day of collection (*P* < 0.05)

Cultured BOEC in P1, P2 and P3-HIGH had similar BDH2 transcript amount compared with oviductal cells in the in vivo state, except in P0 independently from the glucose medium content as well as in P3-LOW, when BDH2 mRNA expression was lower (Fig. 6a). In addition, mRNA expression of BDH2 was not influenced by the different glucose medium content (*P* > 0.05). However, BDH2 transcription rate in BOEC differed through the cell culture passages with a relatively similar pattern in LOW and HIGH glucose medium cultured cells. Oviductal cells in P2-LOW showed almost three-fold higher BDH2 mRNA expression compared with BOEC in P0-LOW and P3-LOW, respectively (*P* < 0.05). In addition, BDH2 mRNA was significantly threefold more highly expressed in cells in P2-HIGH than in cells in P0-HIGH (*P* < 0.05). Oviductal cells collected during the luteal phase showed in P3 cultured with LOW and HIGH glucose medium significant twofold higher (*P* < 0.05) BDH2 transcription than cells harvested during the non-luteal phase, respectively (Fig. 6b and c). Furthermore, oviductal cells collected around ovulation cultured with HIGH glucose medium showed in P0-HIGH a significant higher BDH2 mRNA expression than cells harvested during the luteal phase (Fig. 6c).

A ten- to 20-fold lower OXCT2 mRNA expression was observed in BOEC in vitro in all cell culturing passages compared with the in vivo situation (Fig. 6d). However, oviductal cells showed similar OXCT2 mRNA expression pattern (*P* > 0.05) in different number of cell culture passages (Fig. 6e). Additionally, different glucose medium content did not influence OXCT2 mRNA expression. The OXTC2 transcription rate was in vitro altered by the estrous cycle phase in cultured BOEC in LOW and HIGH glucose medium. In detail, oviductal cells obtained during the luteal phase cultured with LOW glucose medium in P1 displayed an about 20-fold significant higher (*P* < 0.05) OXCT2 mRNA expression than cells harvested during the non-luteal phase (Fig. 6f). In contrast, mRNA expression of OXCT2 in BOEC harvested during the non-luteal phase was significantly higher (*P* < 0.05) in P3-LOW compared with cells collected during the luteal phase. In addition, there was in P2 in presence of HIGH glucose medium an about fivefold higher OXCT2 mRNA expression in BOEC obtained during the luteal phase compared with cells collected during the non-luteal phase (Fig. 6g).

**Fig. 7:**
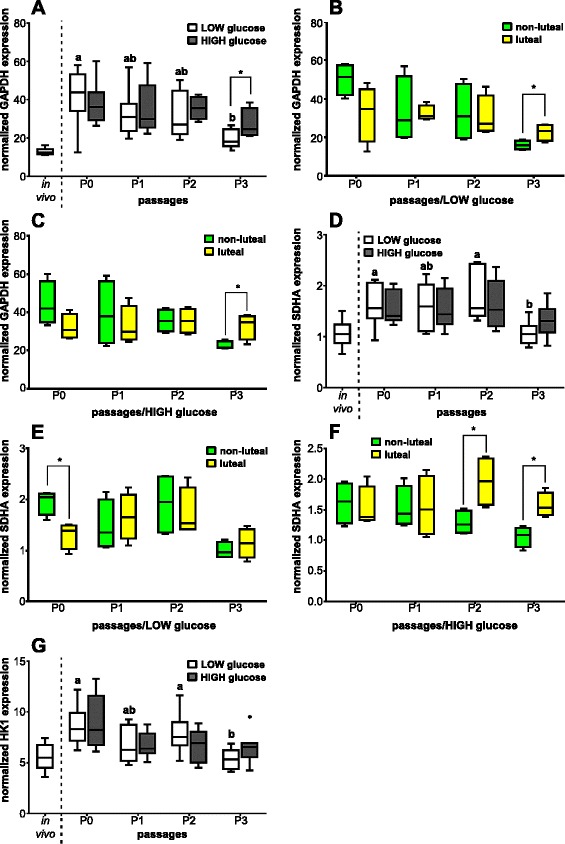
Effect of passage number or estrous cycle stage on GAPDH, SDHA and HK1 mRNA expression. Normalized mRNA expression of **a** GAPDH; **d** SDHA; and **g** HK1 in BOEC of in vivo samples and of cell culture passages P0, P1, P2 and P3 with LOW and HIGH glucose medium content (*n* = 8) as well as normalized mRNA expression depending of the phase of the estrous cycle on day of collecting cells of **b** GAPDH in LOW glucose medium; **c** GAPDH in HIGH glucose medium; **e** SDHA in LOW glucose medium; and **f** SDHA in HIGH glucose medium (*n* = 4). Different letters indicate significant difference (*P* < 0.05) between cell culture passages of the same glucose medium content. Asterisk on top of the line represents significant difference between different glucose content of medium at each cell culture passage or between the different estrous cycle phases on day of collection (*P* < 0.05)

The GAPDH mRNA expression was approximately twofold higher in BOEC in the in vitro situation compared with the in vivo state (Fig. 7a). The number of cell culture passages influenced the GAPDH mRNA amount in the presence of LOW glucose medium but not with HIGH glucose medium. GAPDH mRNA was significantly twofold more highly expressed in BOEC in P0-LOW than in cells in P3-LOW (*P* < 0.05). Furthermore, different glucose medium content altered GAPDH mRNA expression in BOEC showing in P3-HIGH higher contents than in P3-LOW (*P* < 0.05). An approximate twofold increase of GAPDH mRNA expression was observed in P3 in BOEC obtained during the luteal phase cultured with LOW and HIGH glucose medium compared with cells harvested around ovulation, respectively (Fig. 7b and c).

The mRNA expression of SDHA in BOEC in all cell culture passages, except in P3, was about twofold higher compared with the in vivo state (Fig. 7d). The number of cell culture passages had an impact on the transcription rate of SDHA of BOEC cultured in LOW glucose medium but not of cells incubated in HIGH glucose medium. The SDHA mRNA expression in P3-LOW cultured BOEC was significantly lower than in P0-LOW and P2-LOW cells, respectively (*P* < 0.05). Different glucose concentration of the culturing medium did not affect SDHA mRNA expression (*P* > 0.05). The mRNA content of SDHA in P0-LOW cultured BOEC harvested around ovulation was significantly higher (*P* < 0.05) compared with cells obtained during the luteal phase (Fig. 7e). Furthermore, the SDHA mRNA expression in P2 and P3 cultured with HIGH glucose medium in BOEC collected during the luteal phase was significantly higher (*P* < 0.05) than in cells obtained during the non-luteal phase, respectively (Fig. 7f).

The mRNA of HK1 was twofold more highly expressed in oviductal cells in P0 than in BOEC in the in vivo situation (Fig. 7g). However, BOEC in other cell culture passages showed similar HK1 mRNA expression compared with cells in the in vivo state. The cell culture passages altered HK1 transcription in LOW glucose medium cultured cells but not in HIGH glucose medium cultured BOEC. Oviductal cells in P0-LOW and P2-LOW had an about twofold higher HK1 mRNA amount than in P3-LOW cells (*P* < 0.05). Different glucose content of the medium had no impact on HK1 mRNA expression (*P* > 0.05). There was no significant differential regulation of HK1 transcription depending on different phases of the estrous cycle in cultured oviductal cells (data not shown).

The different estrous cycle phase did not affected the mRNA expression of the investigated enzymes of cellular metabolism in the in vivo state in oviductal cells, when comparing samples obtained during non-luteal (pre- and post-ovulatory phase; *n* = 4) with samples from the luteal (*n* = 4) phase (data not shown).

### PGE_2_, OVGP1 and IL8 release from BOEC

The amounts of PGE_2_, OVGP1 and IL8 were estimated in supernatants of BOEC in P0-HIGH and P3-HIGH. These particular cell culture passage numbers and the type of cell culturing medium were chosen based on the mRNA expression results, which showed the most evident differences in the transcription levels of these candidate genes.

**Fig. 8:**
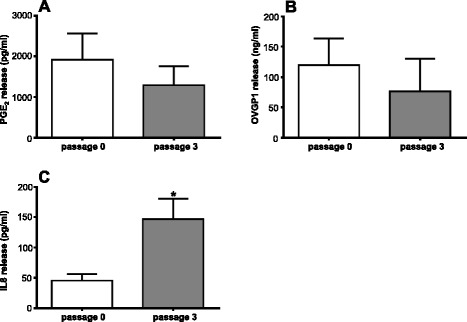
Effect of passage number on PGE_2_, OVGP1 and IL8 release from BOEC. Release estimated in P0-HIGH and P3-HIGH BOEC of **a** PGE_2_ (*n* = 3); **b** OVGP1 (*n* = 3); and **c** IL8 (*n* = 4). Each bar chart illustrates data as means ± SEM. Asterisk depicts significant difference (*P* < 0.05) between P0 and P3

There was no significant change (*P* > 0.05) in PGE_2_ release between P0 and P3 BOEC (Fig. 8a).

Oviductal cells in P0 released higher amounts of OVGP1 (1.5-fold) compared with their cultured counterparts in P3 (Fig. 8b); although this higher release did not reach the significant level.

Cell culture passaging influenced the rate of IL8 release (Fig. 8c). BOEC in P3 released higher amounts of this cytokine (~ three-fold; *P* < 0.05) compared with their counterparts in P0.

## Discussion

Cell culture models are useful tools for revealing specific effects and mechanisms of pure cell populations. However, to obtain valid and reliable information of the treated cells, cell populations should be as close as possible in the in vitro state to the in vivo situation as occur in the organ environment. The present study indicated that each candidate gene differed in its mRNA expression pattern between the in vivo state and the situation in the cultured oviductal cells in vitro until the third passage.

The immunofluorescence staining with cytokeratin as a specific epithelial cell marker revealed that the monolayer cell culturing model within this study contained a pure epithelial cell population throughout all passages as also observed in many other studies [14, 38]. However, it seems very likely that the cell composition of the so called “in vivo” preparation contained as contamination other cell types, e.g. immune cells or fibroblasts. This difference could alter the mRNA expression pattern of cells from the in vivo state to the in vitro situation but does not explain such dramatic change for some genes in their transcription rate.

A possible explanation for the altered mRNA expression rate is the phenomenon described with the term ‘culture shock’ to point out the tremendous stress encountering the primary cells, which leads to higher production of reactive oxygen species (ROS) inducing oxidative stress to the cultured cells [39, 40]. These findings were accompanied by the up-regulation of especially PTGS2 mRNA expression in presence of ROS via the NFk-pathway, which is also likely in the oviduct [18, 41]. One further reason could be due to the stress prompted by hyperglycemia. It has been stated that hyperglycemia strongly induces stress responses specifically in postinjury and postsurgical situations and in systemic inflammatory response syndrome [42], which is similar after tissue collection with disruption of the cells out of their tissue resulting in a higher mRNA expression of PTGS2 and IL8 in this study.

The present study provided a series of data indicating that the mRNA expression of some of the selected genes in BOEC is different starting from P0 throughout the in vitro passages of culturing until P3. More importantly, the release of some of their encoded proteins such as IL8 differed between distinct cell culture passage numbers. This was in accordance with previous investigations, which have shown the consequence of cell culture passages on growth rate, protein level and mRNA expression profile alteration in primary cultured endothelial cells [43, 44]. Especially cells in the LOW glucose medium showed a decrease of mRNA expression of metabolic enzymes. This could be due to the fact that the cell metabolism is reduced and the cells are less proliferative, which is likely in the tissue in most situations when no stimulus is present. Such findings were observed in the untreated controls in vitro [18, 27].

In contrast, specific enzymes for the synthesis of PG were expressed un-regulated throughout all cell culture passages. Constant transcription level of these enzymes along with relatively unchanged release of PGE_2_ indicates that BOEC in this study are functional cells. Prostaglandin E_2_ has been stated the most abundant of the PG in the bovine oviduct conducting a wide range of physiological and immunological functions within this organ [45]. This indicates that the expression of PGE_2_ synthesizing enzymes in cultured BOEC is a functional requirement, which is increased under different physiological treatments, e.g. progesterone, estradiol, cumulus-oocyte-complexes or sperm [17, 18, 27].

Another group of functional proteins are MUC in the oviduct, especially the importance of OVGP1 as the main constitute of oviductal fluid in early embryonic enhancement in different species was described [8, 46, 47]. The WGA staining indicated the presence of mucins on the apical surface of BOEC in vivo, which indicates MUC as an additional functional epithelial cell marker for cell culture approaches, because other cell types were not stained. In addition, a staining was also observed in vitro but the intensity decreased with higher number of cell culture passages, which was accompanied by the decrease of mRNA expression of most MUC in this study. This may be the consequence of oviductal cell dedifferentiation as they go through consecutive subculturing. The anti-adhesive nature of mucins provides the epithelial cells with the first layer of defense mechanism towards pathogens [48]. This alteration of mucins mRNA expression patterns within the in vitro stages of culturing during this study may affect the immune response of cultured BOEC. Additionally, lower expression of OVGP1 at both transcriptome and proteomic level in BOEC cultured in higher cell culture passages could implicate the decrease of secretory granules. The loss of secretory granules in cultured BOEC has been stated [38].

The switch of MUC presence observed in the present investigation is in accordance with studies indicating the effect of in vitro cell culture passage number on metabolic and biochemical activity of primary cells or cell lines from other tissues [49–51]. Halliwell et al. [39] stated that cells, which survive from the ‘culture shock’ induce multiple changes in their metabolic activity and relative metabolic enzyme levels in order to adapt to the new environment. In the present study was observed a higher GAPDH mRNA expression in BOEC incubated with higher glucose medium content. This may result in cells in vitro having metabolic profile far different from their in vivo situation. GAPDH and SDHA mRNA expression decreased by higher number of cell culture passages in the presence of LOW glucose medium content, which within this study supports the statement that these genes are not overall well suited endogenous control genes due to their regulation in different metabolic states [52].

The defined composition of cell culture medium provides good experimental reproducibility in mammalian cell culture. Typical glucose concentration in the culturing medium is 4.5 g/l (25 mM) in the majority of the studies regarding BOEC, which is higher than the blood glucose concentration in bovine of about 0.7 g/l (3.8 mM) [53]. Additionally, this is also higher than the glucose concentration observed in oviductal fluid at different days of the estrous cycle [54]. The actual environment in the cows was simulated for the BOEC in vitro by using cell culture medium containing 1 g/l (5.5 mM) glucose in this study. The mRNA expression data showed that most studied genes, except PTGS2, MUC16, IL8 and GAPDH, had similar pattern in LOW and HIGH glucose medium groups indicating no influence on BOEC in all passages. In this study could be shown for the first time that oviductal cells expressed enzymes for the use of ketone bodies, which would minimize the situation of a negative energy balance when higher ketone bodies were present in the blood [10]. However, the mRNA expression was unexpected independently from the glucose medium content.

Beside the glucose content in the medium, the long term effect of the estrous cycle phase with high progesterone (luteal phase) or high estradiol (around ovulation) contents on BOEC mRNA expression was observed for many investigated factors. Short-time effects of up to 6 h treatment with progesterone or estradiol were observed with up-regulation of PTGS2 mRNA expression in BOEC [27]. However, most of the alterations in mRNA expression pattern occurred in cells cultured at later passages. One explanation might be that subsequent subculturing of BOEC would modify the micro RNA profile regulating present candidate genes mRNA expression. It has been previously mentioned that these noncoding RNA can complement target mRNA, thereby causing translational repression or mRNA degradation [55].

## Conclusion

This study supports the hypothesis that candidate gene mRNA expression patterns in oviductal cells were influenced when they were incubated in the new in vitro environment. Findings from this study might be a good indication of how much in vitro cell culturing alone could alter the mRNA expression of BOEC and to some extent the functionality level of these cells. The obtained results indicate that in vitro situation in general and more importantly the in vitro cell culture passage of the oviductal cells should be carefully considered in studies involving this organ. On the other hand, the application of two different concentrations of glucose in the medium showed that there may be a possibility to culture BOEC with the environment as closely as possible to in vivo. Therefore, researchers conducting experiments with oviductal cells are encouraged considering the impact of cell culture passage number on BOEC response as well as functionality with the advice to use BOEC of P0 when possible.

## Abbreviations

BDH2, 3-hydroxybutyrate dehydrogenase; BOEC, bovine oviductal epithelial cells; cDNA, complementary DNA; DAPI, 4′,6-diamidino-2-phenylindole; GAPDH, glyceraldehyde 3-phosphate dehydrogenase; HDAC1, histone deacetylase 1; HBSS, Hank’s balanced salt solution; HK1, hexokinase 1; IL8, interleukin 8; mRNA, messenger RNA; MUC, mucin; OVGP1, oviduct-specific glycoprotein 1; OXCT2, 3-oxoacid CoA transferase; P, passage; PBS, Dulbecco’s phosphate-buffered saline without Ca^2+^/Mg^2+^; PG, prostaglandin; PPIA, peptidylprolyl isomerase A (cyclophilin A); PTGES2 and −3, prostaglandin E_2_ synthase 2 and 3; PTGS1 and −2, prostaglandin-endoperoxide synthase 1 and 2; RPL19, 60S ribosomal protein L19; SDHA, succinate dehydrogenase complex, subunit A; SUZ12, suppressor of zeste 12 homolog; UXT, ubiquitously-expressed transcript; WGA, wheat germ agglutinin
